# *Porphyromonas gingivalis* secretion leads to dysplasia of normal esophageal epithelial cells *via* the Sonic hedgehog pathway

**DOI:** 10.3389/fcimb.2022.982636

**Published:** 2022-10-03

**Authors:** Xueting Jia, Jinan Liu, Yinxue He, Xiaofeng Huang

**Affiliations:** ^1^ Department of Stomatology, Beijing Friendship Hospital, Capital Medical University, Beijing, China; ^2^ Immunology Research Center for Oral and Systemic Health, Beijing Friendship Hospital, Capital Medical University, Beijing, China

**Keywords:** periodontal-systemic disease interactions, microbiome, *porphyromonas gingivalis*, esophagus, dysplasia, sonic hedgehog

## Abstract

**Objectives:**

To investigate the pathogenic effect of *Porphyromonas gingivalis* cultured media on the esophagus and the mechanism underlying the effect.

**Background:**

Periodontitis is strongly associated with esophageal squamous cell carcinoma (ESCC). The cultured media of *P. gingivalis* may act on healthy esophagus to trigger a malignant transformation; however, this has not been confirmed.

**Methods:**

Cell migration assays and cell cycle measurements were performed on normal human esophageal epithelial cells in the presence or absence of *P. gingivalis* cultured media. The esophagi of healthy adult C57BL/6J mice were isolated and cultured *in-vitro*. Hematoxylin-eosin and immunohistochemical staining using antibodies against proliferating cell nuclear antigen (PCNA), Claudin 1 and Claudin 4 were performed to detect dysplasia in specific tissues. Total mRNA was extracted to determine transcriptional dysregulation. A specific inhibitor of Sonic hedgehog signaling, cyclopamine, was used to confirm the underlying molecular mechanism.

**Results:**

In the presence of *P. gingivalis* cultured media, proliferation and migration of normal human esophageal epithelial cells were up-regulated, and aneuploid cells appeared. Compared with control cells, the arrangement of mouse esophageal epithelial cells became disordered, the percentage of PCNA-positive cells increased, and the positive staining of Claudin 1 and Claudin 4 became weak. In addition, the expression of cancer-related pathway genes was up-regulated but tight junction-related gene expression was down-regulated. The Sonic hedgehog pathway was abnormally activated, and its inhibition reduced the pathogenic effect of *P. gingivalis* cultured media.

**Conclusions:**

We revealed that the cultured media of the key periodontal pathogen, *P. gingivalis*, can induce the malignant transformation of normal esophageal epithelium through the Sonic hedgehog pathway.

## 1 Introduction

Periodontitis is a common oral inflammatory disease with a high prevalence of 45% to 50% and is the sixth most common human disease. The average worldwide prevalence of severe periodontitis has been estimated to be 11% (Kassebaum et al., 2014; Slots, 2017; Sanz et al., 2020) and recent studies support association between periodontitis and several systemic diseases, including diabetes, cardiovascular diseases, chronic obstructive pulmonary disease, and digestive cancers, such as esophageal cancer (Chapple et al., 2013; Linden et al., 2013; Zhou and Lou, 2019; Sanz et al., 2020).

Esophageal cancer ranks as the eighth most common cancer worldwide and the 5-year overall survival rate is only 10% because of its aggressive nature and the difficulty of early diagnosis (Pennathur et al., 2013; Domper Arnal et al., 2015; Gupta and Kumar, 2017; Zhou and Lou, 2019). Esophageal cancer consists of two main histological subtypes, esophageal squamous cell carcinoma (ESCC) and esophageal adenocarcinoma (EAC). In developing countries, ESCC is the predominant subtype. Epidemiological studies show that patients with periodontitis may have a significantly higher risk of ESCC, even after controlling for confounding variables, such as smoking (Abnet et al., 2001; Hiraki et al., 2008; Chen et al., 2015; Chen et al., 2016; Nwizu et al., 2017). These findings indicate that oral bacterial infections may be significantly related to ESCC susceptibility.

*Porphyromonas gingivalis*, one of the “red complex” bacterial species in the oral cavity, is a representative pathogen of periodontitis. Accumulating evidence indicates that *P. gingivalis* is strongly associated with ESCC. The abundance of *P. gingivalis* in saliva and dental plaque is associated with an increased occurrence and poor prognosis of ESCC, and high levels of *P. gingivalis*-specific antibodies in serum is an independent risk factor for poor ESCC prognosis (Ahn et al., 2012; Peters et al., 2017; Gao et al., 2018; Chen et al., 2019; Kageyama et al., 2019; Meng et al., 2019; Chen et al., 2021; Kawasaki et al., 2021). Additionally, the detection rates of *P. gingivalis* antigen, the secreted gingipain protease, and specific 16S rDNA are significantly higher in ESCC tissue compared with rates in adjacent tissue or in healthy controls (Gao et al., 2016; Yuan et al., 2017). Few studies have investigated the mechanism for the association between *P. gingivalis* and ESCC. Based on studies of ESCC patients, animal cancer models, and ESCC cell lines, *P. gingivalis* promotes the progression of the malignant phenotype and reduces therapeutic efficacy. The chronic inflammatory response, immune invasion, and epithelial-mesenchymal transition represent key links between the presence of *P. gingivalis* and ESCC development (Yuan et al., 2019; Qi et al., 2020; Chen et al., 2021).

However, the studies above focused on the effect of *P. gingivalis* on damaged esophagus in which cancer was already established. It is not known whether the cultured media of *P. gingivalis* can affect healthy esophageal epithelium and trigger dysplasia. Therefore, the aim of this study was to investigate the effect of *P. gingivalis* cultured media on healthy esophagus and to explore the underlying mechanism of any effect.

## 2 Materials and methods

### 2.1 Preparation of *P. gingivalis* strain cultured media

*P. gingivalis* liquid medium consisted of 30g/L tryptone soybean broth, 1 mg/mL yeast extract, 1 mg/L vitamin K1 and 5 mg/L hemin. For culture plates, 0.2 g/L agar and 5% sterile defibrinated sheep blood were added.

A standard strain of *P. gingivalis*, ATCC 33277 (kindly provided by Prof. Huanxin Meng, Peking University School and Hospital of Stomatology, China), was inoculated on plates and cultured at 37°C under anaerobic conditions (AnaeroPouch, MGC, Japan). A monoclonal colony was picked and amplified in the liquid medium. When the bacteria grew to the exponential phase, a density of 0.6 (OD_600_), the bacterial suspension was centrifuged at a speed of 10,000 × g for 10 minutes at 4°C to collect the cultured media. The cultured media was filtered through a 0.22-μm filter (Millipore, Ireland) before adding to cell or organ culture media at the required concentration.

### 2.2 Cell culture

Normal human esophageal epithelial Het-1A cells were cultured in Roswell Park Memorial Institute (RPMI) 1640 medium (Invitrogen, USA) supplemented with 10% fetal bovine serum (FBS, Thermo Fisher, USA) at 37°C in 5% CO_2_.

### 2.3 Cell migration assay

Het-1A cells were assigned to three groups: cultured in medium supplemented with 5% *P. gingivalis* blank medium (0% CM), cultured in medium supplemented with 0.5% *P. gingivalis* cultured media (0.5% CM), and cultured in medium supplemented with 5% *P. gingivalis* cultured media (5% CM). After culture for 24 h, Het-1A cells from each group were trypsinized and resuspended in 500 μL serum free medium, and seeded at a density of 2 × 10^5^ cells/mL in the upper wells of transwell inserts with a polyethylene terephthalate membrane pore size of 8.0 µm (MCEP24H48, Millipore, USA) in a 24-well plate. The lower chamber contained 750 μL culture medium containing 10% FBS. Cells were cultured in the transwell at 37°C with 5% CO_2_ for 24 hours. Then, culture medium was replaced by PBS in both chambers. After that, cells attached on the upper surface of the membrane were wiped off with a cotton swab, and cells that had migrated across the transwell membrane were fixed with methyl alcohol for 5 minutes, stained with DAPI, and counted under a fluorescence microscope.

### 2.4 Cell cycle measurement

The cell cycle of esophageal epithelial cells stained by propidium iodide (PI) was analyzed by flow cytometry. Het-1A cells (1×10^6^ cells) were digested with 0.25% trypsin to make a single cell suspension, washed twice with PBS, and then resuspended in 250 μL PBS. The cells were then fixed overnight at 4°C by the addition of 750 μL anhydrous ethanol. The next day, the cells were centrifuged and washed with PBS. One milliliter of DNA staining solution (containing 0.2 mg/ml RNase, Cell Cycle Staining Kit, MultiSciences, China) was added to 10^6^ cells, vortex-mixed for 5 to 10 seconds and incubated in the dark at room temperature for 30 minutes. The lowest sample loading speed was selected for the flow cytometer (Attune NxT, Thermo Fisher Scientific, USA). Flow cytometry was used to examine the cell cycle in accordance with standard procedures. Cell cycle distribution was analyzed by ModFit, and the proportions of cells in G0/G1, S and G2/M were calculated.

### 2.5 Organ culture *in vitro*

Fifteen healthy adult C57BL/6J mice were kept in the Institute of Materia Medica, Chinese Academy of Medical Sciences & Peking Union Medical College under standard conditions, and randomly assigned to the following groups: “0% CM” (treated with 5% *P. gingivalis* blank medium as control, n = 3), “0.5% CM” (treated with 0.5% *P. gingivalis* cultured media, n = 3), “5% CM” (treated with 5% *P. gingivalis* cultured media, n = 3), “0.5% CM + cyclopamine (CPM)” (treated with 0.5% *P. gingivalis* cultured media and 5 μM cyclopamine to inhibit SHH signaling (CPM, MCE, USA), n = 3) and “5% CM + CPM” (treated with 5% *P. gingivalis* cultured media and 5 μM CPM, n = 3). At a minimum, biological triplicates were analyzed. The organ culture system was described previously (Jia et al., 2018).

### 2.6 Immunohistochemical and immunofluorescence staining

Sections were prepared as previously reported (Jia et al., 2018). Antibodies against the following were used for immunohistochemical analysis: proliferating cell nuclear antigen (PCNA, mouse or rabbit monoclonal; 1:50, Santa Cruz Biotechnology), Claudin 1 (CLDN1, Proteintech), Claudin 4 (CLDN4, Proteintech), SHH (1:50, Abcam), GLI1 (1:50, Abcam). Corresponding secondary antibodies were used (1:100, ZSGB-BIO). For immunohistochemical staining, reactions were developed with diaminobenzidine (DAB, ZSGB-BIO). The percentage of PCNA positive cells per 400 times microscopic field was counted in a minimum of 3 sections. The Image J software calculates the staining area and the percentage of positive area of CLDN1, CLDN4 and SHH staining sections. For immunofluorescence staining, the sections were incubated with fluorescent secondary antibodies [Fluorescein-Conjugated AffiniPure Goat Anti-Mouse IgG (H+L), ZF-0312, ZSGB-BIO, China] at 37°C for 1 hour in the dark, washed with PBS, mounted with fluorescent mounting medium containing DAPI (ZLI-9557, ZSGB-BIO, China) and observed by fluorescence microscopy (FV1000, Olympus, Japan).

### 2.7 RNA isolation and sequencing

Total RNA from Het-1A cells of the 0% CM and 5% CM groups was extracted and purified in accordance with the manufacturer’s protocol. Sequencing libraries were generated using the VAHTS mRNA-seq v2 Library Prep Kit for Illumina in accordance with the manufacturer’s recommendations, and index codes were added to attribute sequences to each sample. The libraries were sequenced on an Illumina NovaSeq platform to generate 150-bp paired-end reads following the manufacturer’s instructions. Raw data (raw reads) were first processed through primary quality control to obtain clean data (clean reads), which were used for downstream analyses. Paired-end clean reads were aligned to the reference genome using TopHat. HTSeq was used to count the reads mapped to each gene. Differential expression analysis between two conditions was performed using the DEGSeq R package (1.20.0). Differentially expressed genes (DEGs) were defined as those for which the adjusted P-value was below 0.05 and the log2(fold change) was more than 1. Gene Ontology (GO) and KEGG enrichment analyses of DEG sets were performed using GOseq R and KOBAS 3.0, respectively. GO terms with adjusted P-value below 0.05 were considered significantly enriched by DEGs.

### 2.8 Real-time quantitative polymerase chain reaction

Total RNA was isolated from Het-1A cells of 0% CM, 0.5% CM, and 5% CM groups using TRNzol (DP424, TIANGEN BIOTECH, China) in accordance with the manufacturer’s instructions. First-strand cDNA was synthesized using the RevertAid First Strand cDNA Synthesis Kit (K1622, Thermo, USA) in accordance with the manufacturer’s protocol. SYBR^®^ Premix Ex Taq™ II (Tli RNase H Plus, RR82LR, TaKaRa) was used to perform RT-qPCR. The qPCR thermocycling conditions were: 95°C for 5 min; 40 cycles of 95°C for 10 seconds, and 60°C for 35 seconds. After the amplification reaction, 70 cycles (60°C to 95°C for 10 sec) were performed to establish the melting curve of the PCR product. The 2^−ΔΔCt^ method was applied to calculate relative mRNA levels. The primer sequences used in the study were: 1) *GAPDH* forward, 5′-AAATCAAGTGGGGCGATGCT-3′ and reverse, 5′-CAAATGAGCCCCAGCCTTCT-3′; 2) *HHIP* forward, 5′-AATGCAGAGCCACGGTACAA-3′ and reverse, 5′-CAGAGGCACTTGTTCGGTCT-3′.

### 2.9 Statistical analysis

Statistical analyses were performed using SPSS software version 25. Data are expressed as means ± SD of at least three independent experiments. The conduct of the experiment and the data assessment were performed by different researchers. Statistical differences of PCNA, SHH, CLDN1, CLDN4 positive rates and migration cell numbers among groups were analyzed by one-way ANOVA. The CLDN1/CLDN4 positive area percentages were compared between the samples with CPM addition and the samples without blocking the Shh pathway by paired t-test. *P* values below 0.05 were considered statistically significant.

## 3 Results

### *3.1 P. gingivalis* cultured media leads to malignant transformation of esophageal epithelial cells

#### 3.1.1 Cell culture results

**Figure 1:**
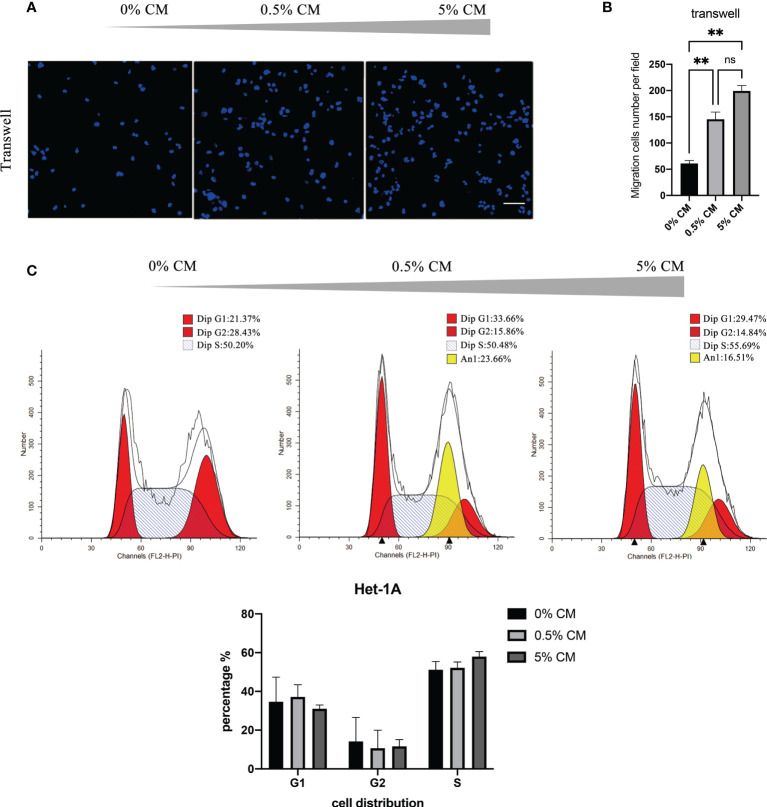
P*. gingivalis* cultured media promotes migration and proliferation of Het-1A cells. **(A)** Pictures of typical transwell assays. **(B)** Statistical analysis of transwell assay results. **(C)** Cell cycle analysis with propidium iodide staining. Scale bar: 100 μm. **P < 0.01, ns, no statistical significance.

The mean numbers of cells that migrated across the transwell membrane were significantly different among groups (61 cells in the 0% CM group, 145 cells in the 0.5% CM group, and 199 cells in the 5% CM group, *P* < 0.0001). Representative pictures and the corresponding statistics of transwell assays are presented in **Figures 1A, B**. The number of migration cells increased significantly in 0.5% CM group than in the 0% CM group (*P* = 0.0098). Similarly, the number of migration cells in 5% CM group was also significantly higher than that in 0% CM group (*P* = 0.0054). The effect of *P. gingivalis* cultured media on cell cycle progression was also determined. The addition of *P. gingivalis* cultured media for 24 h did not change the proportion of cells in S phase (still 50% approximately); however, a large number of aneuploid cells appeared as shown in **Figure 1C**. The aneuploid cells accounted for 23.66% of cells in the 0.5% CM group and for 16.51% in the 5% CM group (**Figure 1C**). The cell cycle distribution did not show statistically significant differences among groups (*P* > 0.05).

#### 3.1.2 Organ culture results

**Figure 2:**
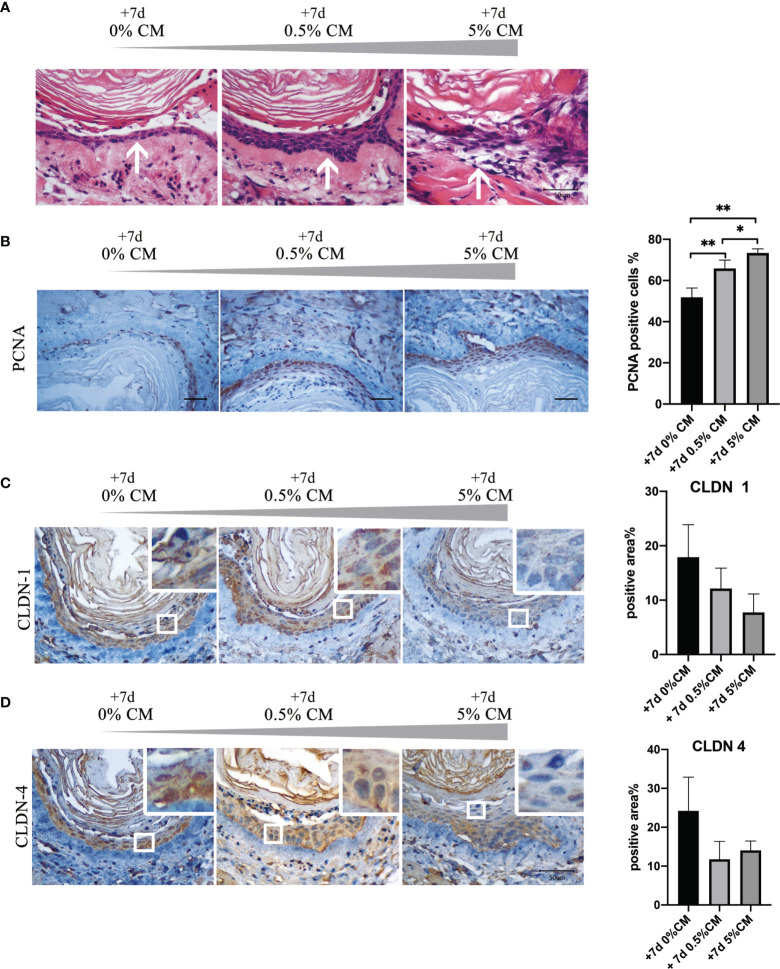
Addition of *P. gingivalis* cultured media to normal mouse esophagus *in vitro* for 7 days leads to malignant transformation. **(A)** The number of esophageal epithelial cell layers increased after treatment with 0.5% and 5% *P. gingivalis* cultured media and the cell arrangement was disordered in the 5% CM group (white arrows). **(B)** The rate of PCNA-positive cells was significantly in 0.5% CM and 5% CM groups compared with the control group. **(C, D)** The expression of CLDN1 and CLDN4 was down-regulated gradually as the concentration of *P. gingivalis* cultured media increased compared with the control esophageal epithelium. Scale bar: 50 μm. *P < 0.05; **P < 0.01.

Treatment of normal mouse esophagus with *P. gingivalis* cultured media for 7 days significantly increased the number of stratified epithelial cell layers and the cell arrangement became disordered as shown in **Figure 2A** (white arrows). A statistically significant increase in the percentage of PCNA-positive cells was detected, from 51.8% to 65.9% in the 0.5% CM group (*P* = 0.010). In the 5% CM group, this percentage increased even further to 73.4% (*P* = 0.005, **Figure 2B**). PCNA results indicated that cellular proliferative capacity was increased by *P. gingivalis* cultured media.

However, the expression of the cell tight junction markers, CLDN1 and CLDN4, decreased with the increase in *P. gingivalis* cultured media concentration as shown in **Figure 2C**, indicating esophageal epithelial barrier was impaired. The percentage of positive area in CLDN1 immunohistochemical staining section was much lower in 5% CM group, although the statistically significant difference did not exist (*P* = 0.066). With respect to CLDN4, the percentage of positive area was also much lower with the addition of *P. gingivalis* cultured media (0% CM group vs. 0.5% CM group, *P* = 0.093; 0% CM group vs. 5% CM group, *P* = 0.197).

### 3.2 RNA-seq data indicated up-regulation of genes related to cancer and down-regulation of genes related to tight junctions

**Figure 3:**
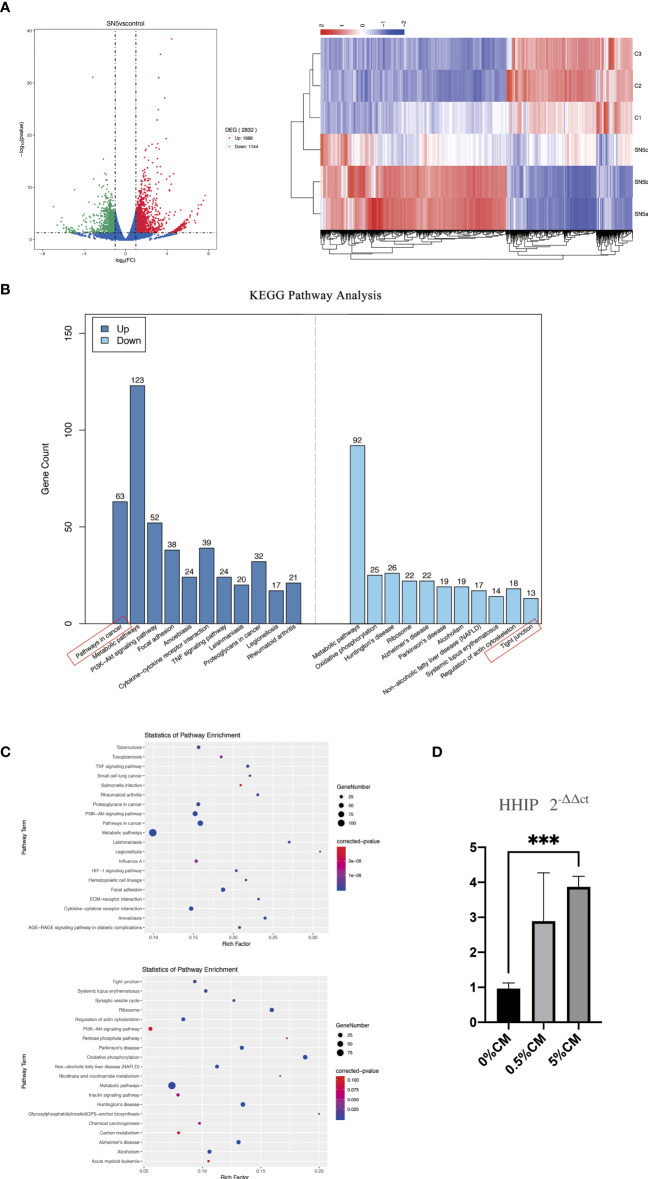
Comparison of the transcriptome profiles of cultured esophagi from 0% CM and 5% CM groups. **(A)** Volcano plot presenting the number, fold change, and statistical significance of DEGs and the cluster analysis of DEGs. Rows represent samples, and columns represent genes. **(B)** Up- and down-regulated pathways according to KEGG pathway analysis. **(C)** Numbers of DEGs and corresponding *P* values in terms related to up- and down-regulated pathways. **(D)** Differences in *HHIP* expression among 0% CM, 0.5% CM and 5% CM groups were determined by RT-qPCR. ***P < 0.001.

To profile global gene expression related to the malignant transformation of esophageal cells after treatment with *P. gingivalis* cultured media, total mRNA of Het-1A cells from the 0% CM and 5% CM groups was extracted and sequenced. A total of 2832 DEGs were detected between 0% CM and 5% CM groups. Addition of 5% *P. gingivalis* cultured media for 24 hours promoted overexpression of 1688 genes and down-regulated expression of 1144 genes (**Figure 3A**).

KEGG pathway analysis showed the primary up-regulated pathway to be in cancer, followed by metabolic pathways, PI3K-Akt signaling, focal adhesion, amoebiasis, cytokine-cytokine receptor interaction, and TNF signaling. The pathway associated with tight junctions was down-regulated (**Figure 3B**). The expression levels of genes associated with the cancer pathway, such as *WNT3A*, *WNT2B*, *PTK2*, *PTGS2*, *CSF1R*, *ITGA2*, *MMP9*, *LPAR3*, *LPAR1*, *MMP2* and Hedgehog-interacting protein (*HHIP*) were up-regulated. Among these up-regulated genes, *HHIP* attracted our attention because it is a target gene of the SHH pathway, which is silent in normal mature esophageal cells, but is closely linked to cell proliferation and mobility. Up-regulation of *HHIP* was confirmed by RT-qPCR, *P* < 0.0001.

**Figure 4:**
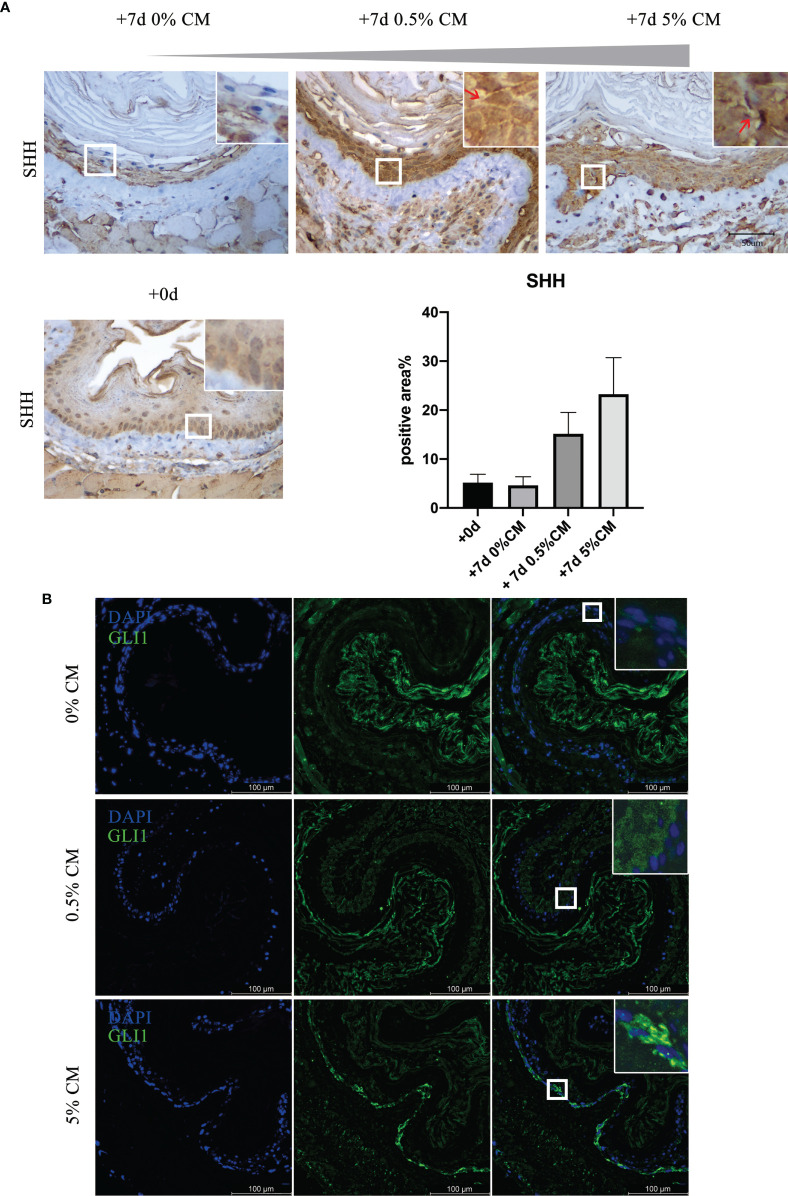
The SHH pathway was activated in *P. gingivalis* cultured media-treated mouse esophagi. **(A)** Overexpression of SHH protein in epithelial cells in 0.5% CM and 5% CM groups as indicated by red arrows. **(B)** GLI1 expression increased with 0.5% or 5% *P. gingivalis* cultured media treatment for 7 days, and the GLI protein was translocated from the cytoplasm into the nucleus of epithelial cells, especially in the 5% CM group. Scale bars: 50 μm **(A)**; 100 μm **(B)**.

We then examined the expression of SHH of *in vitro*-cultured mouse esophagus. Immunohistochemistry indicated that, in normal mouse esophageal epithelial cells, SHH was nearly absent. However, with *P. gingivalis* cultured media treatment, strong positive expression of SHH was detected (**Figure 4**, red arrows). Compared with expression at the time of sacrifice and 7 days after culture, the amount of SHH positive staining increased markedly in 0.5% CM group (+7d 0% CM group vs. +7d 0.5% CM group, *P* = 0.0929) and in 5% CM group (+7d 0% CM group vs. +7d 5% CM group, *P* = 0.1421), although the statistically significant difference did not exist. Consistent with SHH expression pattern, the expression levels of GLI1, a marker for Hh-responding cells, were barely detectable in *in vitro*-cultured mouse esophagus. With *P. gingivalis* cultured media treatment for 7 days, GLI1 expression became positive and was transported from the cell cytoplasm into the nucleus, which is critical to indicate activation of the SHH pathway.

### 3.3 Inhibition of the SHH pathway reduces the pathogenic effect of *P. gingivalis* cultured media

**Figure 5:**
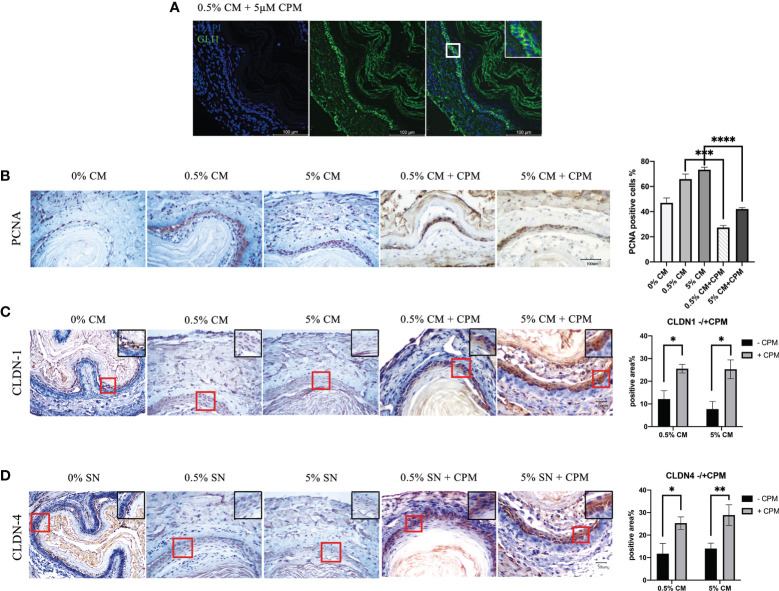
SHH inactivation reduced the percentage of PCNA-positive cells, which was increased in esophagi by *P. gingivalis* cultured media treatment, and recovered the expression of tight junction markers. **(A)** Predominately cytoplasmic localization of GLI1 in mouse esophageal epithelial cells cultured in CPM-supplemented medium. **(B)** Percentages of PCNA-positive cells in 0.5% CM and 5% CM groups were significantly decreased when CPM was added. **(C)** CLDN1 expression was decreased in 0.5% CM and 5% CM groups and was increased back to normal in the esophageal epithelium with the addition of CPM. **(D)** Accumulation of CLDN4 in esophageal epithelial cells in the presence of CPM, compared with culture without CPM. Scale bars: 100 μm **(A)**; 50 μm **(B–D)**. *P < 0.05; **P < 0.01; ***P < 0.001; ****P < 0.0001.

To investigate the role of SHH during the malignant transformation of normal esophageal epithelium by treatment with *P. gingivalis* cultured media, 5 μM of a specific SHH signaling inhibitor, CPM, was added to the culture medium for 7 days. GLI1 protein was predominately localized in the cytoplasm in samples treated with *P. gingivalis* cultured media plus CPM (**Figure 5A**), indicating disturbed *Shh* signaling. In the 0.5% *P. gingivalis* cultured media-treated samples, the percentage of PCNA-positive cells decreased significantly with the addition of 5 μM CPM (*P*<0.001), and this difference also existed between 5% CM and 5% CM + CPM groups (*P*<0.0001, **Figure 5B**).

In addition, CLDN1 expression in the esophageal epithelial cells became markedly positive in the presence of CPM (0.5% CM group vs. 0.5% CM + CPM group, *P* = 0.027; 5% CM group vs. 5% CM + CPM group, *P* = 0.027; **Figure 5C**). The percentage of positive area in CLDN4 immunohistochemical staining section was also significantly higher in the presence of CPM (0.5% CM group vs. 0.5% CM + CPM group, *P* = 0.026; 5% CM group vs. 5% CM + CPM group, *P* = 0.009; **Figure 5D**). The expression patterns of CLDN1 and CLDN4 indicated recovery of tight junctions and intactness of the epithelial barrier after blocking the SHH pathway in esophageal epithelial cells.

## 4 Discussion

Transition of the esophagus from a normal to a cancerous state has been intensively investigated. ESCC arises in a sequential manner, beginning with inflammation, flowed by hyperplasia, dysplasia, carcinoma *in situ*, and invasive carcinoma (Chen et al., 2017). Accumulating evidence supports a close correlation between periodontal pathogens and esophageal carcinoma stage. In a prospective study, bacteria in oral wash samples were assessed. After adjusting for body mass index, smoking and alcohol habits, the abundance of *P. gingivalis* marginally trended with a higher risk of ESCC (OR = 1.30; P = 0.09) (Peters et al., 2017). Analysis of 190 ESCC patients for a median follow-up of 5-years showed that *P. gingivalis* in the cytoplasm of ESCC cells and stroma was positively correlated with invasion depth, lymphatic metastasis, tumor-node metastasis stage, and shorter overall survival (Qi et al., 2020).

The molecular mechanism underlying the intracellular invasion of *P. gingivalis* to promote the aggressive progression of ESCC is poorly understood. Qi et al. revealed that activation of TGFβ-dependent Smad/YAP/TAZ signaling may be involved (Qi et al., 2020). Meng et al. found that enhanced proliferation and motility of ESCC cells induced by *P. gingivalis* infection *in vitro* were accompanied by activation of NF-κB signaling (Meng et al., 2019). Moreover, Chen et al. found that *P. gingivalis* could up-regulate the glucose uptake rate of ESCC cells and increase the incidence of invasive carcinoma in a mouse esophageal tumor model induced by 4-nitroquinoline 1-oxide. In addition, *P. gingivalis* can also promote epithelial-mesenchymal transition and the recruitment of myeloid suppressor cells by up-regulating interleukin 6 (IL6) (Chen et al., 2021). Yuan et al. identified epigenetic alteration in ESCC patients with intracellular *P. gingivalis* infection, and blocking B7-H4 LD5B could improve the protective immunity against *P. gingivalis* (Yuan et al., 2019).

However, almost all of the above studies focused on the effect of *P. gingivalis* in the carcinoma phase, not on earlier stages of the oncogenesis process, which has important clinical significance. In this study, we paid close attention to the causal relationship between the cultured media of *P. gingivalis* and the squamous dysplasia stage, which is a critical pathological stage during the oncogenesis process of ESCC.

Dysplasia is defined by the histopathological appearance of nuclear atypia, loss of normal cell polarity, and abnormal tissue maturation without invasion through the basement membrane (Dawsey et al., 1994; Dawsey et al., 1994). In our study, *P. gingivalis* cultured media promoted the proliferation and migration of normal esophageal epithelial Het-1A cells. More importantly, aneuploid cells appeared after treatment for 24 h (**Figure 1**). Additionally, after treatment of normal mouse esophagus with *P. gingivalis* cultured media *in vitro*, the number of epithelial cell layers increased, the cell arrangement became disordered, the rate of cells positive for the proliferation marker, PCNA, was significantly increased, and the positive staining of the tight junction markers, CLDN1 and CLDN4, became weak (**Figure 2**). Consistent with this phenotype, KEGG pathway analysis of human esophageal epithelial cell transcriptome sequencing showed that after treatment with *P. gingivalis* cultured media, cancer pathway-related genes were up-regulated, and tight junction pathway-related genes were down-regulated (**Figure 3**).

These results indicated that *P. gingivalis* cultured media can directly induce dysplasia of esophageal epithelial cells. This important finding may explain why a high abundance of *P. gingivalis* in saliva has an increased risk of ESCC; that is, before epithelial barrier destruction, the harmful cultured media has already been acting on the healthy esophagus (Peters et al., 2017). In 2020, Qi et al. suggested that *P. gingivalis* alone might not be tumorigenic during the onset of ESCC because NE6 cells, an immortal cell line, failed to form colonies in soft agar colony formation assays after *P. gingivalis* infection and because subcutaneous xenografts from *P. gingivalis*-infected NE6 cells grew slowly and gradually disappeared (Qi et al., 2020). Our findings do not exactly correspond to those of Qi et al.; however, this difference is reasonable because of the complicated influence of many factors and the long process from the dysplasia stage to the carcinoma stage.

In this study, esophageal epithelial dysplasia was accompanied by activation of the SHH pathway, as shown in **Figures 3**, **4**. *SHH* is a significant embryonic development-related gene, which is exclusively expressed in epithelial cells. The encoded SHH protein acts on mesenchymal cells through a paracrine pathway. A large number of studies showed the SHH pathway to be silent in normal mature esophageal epithelial cells, but in dysplasia and cancer, the SHH pathway is activated (Zhu et al., 2011; Okumura et al., 2014; Wadhwa et al., 2017). The SHH pathway inhibitor, CPM, has been used in the clinical treatment of cancer (Berman et al., 2003; Yu et al., 2019). We previously confirmed that SHH can regulate the proliferation and differentiation capacity of mouse esophageal epithelial cells. In *K14-Cre;Shh^fl/fl^* mice, the number of esophageal epithelial cells decreased and proliferation was disrupted compared with wild-type littermates. There were also significant differences in the expression of genes related to the cell cycle and cell motility between mutant and control samples, indicating a relationship of the SHH pathway with malignant transformation (Jia et al., 2018).

To further investigate the role of SHH activation in the *P. gingivalis* cultured media-induced phenotype, we added 5 μM CPM to the culture medium to block SHH signaling (**Figure 5A**). In the CPM-treated samples, *P. gingivalis* cultured media failed to increase PCNA-positive rates or to break down tight junctions (**Figure 5**), indicating that the SHH pathway may be the molecular mechanism underlying *P. gingivalis* cultured media-induced esophageal epithelial dysplasia. This finding may be of significance in the search for new targets to prevent esophageal epithelial dysplasia. Further studies are required to confirm the pathogenic effect *in vivo* and to identify the key pathogenic components in *P. gingivalis* cultured media.

## 5 Conclusions

We show that the cultured media of the key periodontal pathogen, *P. gingivalis*, can initiate the malignant transformation of normal esophageal epithelium through the SHH pathway. Our study provides direct causal evidence for the carcinogenic effect of a periodontal pathogen on normal esophagus. These findings will help identify new therapeutic targets for ESCC and inform the development of measures for the early prevention of ESCC.

## Data availability statement

The data presented in the study are deposited in the Gene Expression Omnibus repository, accession number GSE210408.

## Ethics statement

The animal study was reviewed and approved by the Biomedical Ethics Committee, Beijing Friendship Hospital, Capital Medical University.

## Author contributions

XJ and XH conceived the study and designed the research; XJ, JL, and YH performed the experiments; JL and YH contributed to data analysis and interpretation; XJ and XH wrote the manuscript; XH contributed to manuscript review. All authors contributed to the article and approved the submitted version.

## Funding

This work was supported by the National Natural Science Foundation of China (to XJ, No. 82101005), the National Natural Science Foundation of China (to XH, No. 82071141), and the Natural Science Foundation of Beijing Municipality (to XH, No.7202036).

## Acknowledgments

We thank Prof. Huanxin Meng (Peking University School and Hospital of Stomatology, China) for the generous gift of the standard strains of *P. gingivalis*, ATCC 33277. We acknowledge Xiaoqian Xu (Beijing Friendship Hospital, Capital Medical University, China) for assistance on the statistical analysis. We thank Jeremy Allen, PhD, from Liwen Bianji (Edanz) (www.liwenbianji.cn) for editing a draft of this manuscript. In addition, the authors would like to appreciate Qiping Luo for his kindly support on figure preparation.

## Conflict of interest

The authors declare that the research was conducted in the absence of any commercial or financial relationships that could be construed as a potential conflict of interest.

## Publisher’s note

All claims expressed in this article are solely those of the authors and do not necessarily represent those of their affiliated organizations, or those of the publisher, the editors and the reviewers. Any product that may be evaluated in this article, or claim that may be made by its manufacturer, is not guaranteed or endorsed by the publisher.
